# Spinal dysraphism

**DOI:** 10.11604/pamj.2020.37.146.26288

**Published:** 2020-10-13

**Authors:** Vinoth Kumar Perumal, Krishnaprasanth Baalann

**Affiliations:** 1 Department of Community Medicine, Sree Balaji Medical College and Hospital, Bharath Institute of Higher Education and Research Institute, Chennai, India

**Keywords:** Neural tube defect, folate deficiency, antenatal visits

## Image in medicine

Defects of the spinal cord results from abnormal closure of the neural folds in the earlier weeks of development. If closure fails anywhere from the cervical region to caudal region it is called as spina bifida, most commonly involving the lumbosacral region. Meningocele is the simplest form of neural tube defect characterised by cystic dilation of meninges containing cerebrospinal fluid without any neural tissue. In India, it affects about 1.9 per 1000 births. Not having enough folate (vitamin B9) in the diet plays a significant role besides genetic factor, obesity, poorly controlled diabetes and anti-seizure medications. Standard treatment is surgery after birth. We present a case of a newborn female baby, term normal vaginal delivery, with normal birth weight, with a mass protruding from lower back region. Baby´s mother gave antenatal history of failing to take folate (vitamin B9) and irregular visits to the hospital. On examination, a small, moist, spherical sac measuring roughly 4 x 4 cm, protruded through the gap in the spine. This sac contained a portion of the spinal cord membrane (meninges) and some spinal fluid. Baby was examined thoroughly for any other abnormalities. Baby was planned for meningocele repair on the same day as there were no other complications. Post-natal advice and counselling to protect child´s skin, preventing sores, special diet to prevent obesity, was given to the mother and discharged the next day. Child was referred to a neurologist and urologist for further management and prevent any complications that may arise.

**Figure 1:**
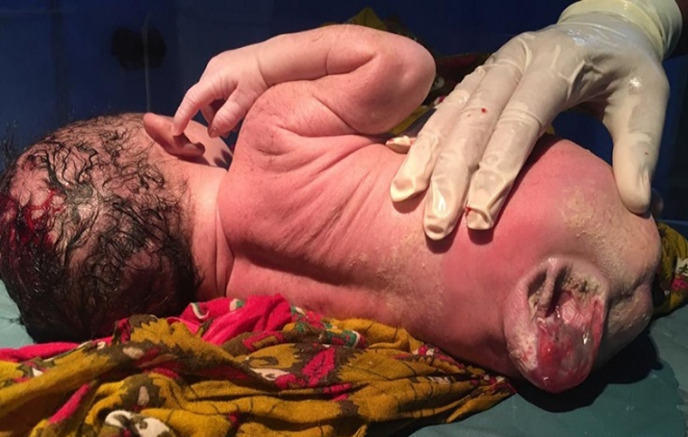
meningocele

